# Validation of uterine artery embolization before surgical laparoscopic myomenucleation compared to single surgical laparoscopic myomenucleation for the treatment of large fibroids and uterus myomatosus

**DOI:** 10.3389/fmed.2023.1145952

**Published:** 2023-04-17

**Authors:** Stefan Hertling, Ekkehard Schleußner, lsabel Graul

**Affiliations:** ^1^Department of Obstetrics, Jena University Hospital, Jena, Germany; ^2^Department of Orthopedics, Jena University Hospital, Eisenberg, Germany; ^3^Department of Trauma, Hand and Reconstructive Surgery, Jena University Hospital, Friedrich Schiller University Jena, Jena, Germany

**Keywords:** uterus, uterus myomatosus, big uterine fibroids, uterine embolization, laparoscopic myoma enucleation

## Abstract

**Aim:**

To determine the efficacy of preoperative uterine artery embolization (uterine artery embolization; UAE) prior to elective laparoscopic fibroid removal compared to single laparoscopic fibroid removal in women with large uterine fibroids and women with uterus myomatosus.

**Material and methods:**

A total of 202 women with symptomatic uterine fibroids who were scheduled for elective fibroid enucleation were included in this retrospective, monocentric, non-randomized study. Two procedures were compared: women who received percutaneous UAE 24 h prior to elective laparoscopic fibroid eviction for large uterine fibroids (>6 cm) and uterus myomatosus. And women who received laparoscopic fibroid enucleation alone for large uterine fibroids and uterus myomatosus. Outcome parameters for effectiveness were the hospital stay, the operating time and the intraoperative blood loss.

**Results:**

Women who underwent preoperative percutaneous embolization of the uterine arteries, both for large fibroids and uterus myomatosus, had significantly less blood loss, shorter hospital stays, and shorter operating times.

**Conclusions:**

Especially women with large uterine fibroids and women with uterus myomatosus after having children can benefit from the combination therapy of preoperative percutaneous uterine embolization with subsequent laparoscopic myoma enucleation.

## Introduction

“The only definitive therapy for symptomatic uterine myomatosus is hysterectomy” (1).

This statement no longer corresponds to current treatment recommendations and surgical practice in surgical gynecology. More and more patients explicitly want the uterus to be preserved. Several national and international studies reflect this trend (2, 3). Uterine transplants are among the modern surgical procedures. The first living donation was made in 2013. Until a few years ago, such an operation was unthinkable. Only a very limited number of successful uterine transplants can be found in the literature. This shows how important it is to establish modern surgical procedures and how important uterine-preserving surgical measures are (4). From a health economic point, fibroids cause high treatment costs. A study from the USA estimates health costs in the amount of several billions of US dollars per year and, together with absenteeism and obstetric complications, the costs add up to several tens of billions per year in women's health (5). Therefore, the development of effective treatment options for the most common benign disease in women over 35 years of age (6) is important. The combination of uterine artery embolization (UAE) and conservative myomectomy is an established treatment option in the surgical treatment of fibroids. By reducing intraoperative blood loss, UAE facilitates surgery and improves the chances of surgical myoma enucleation. This provides a better starting point for performing a uterine-preserving surgical myomectomy. Reconstruction of the uterus is simplified (7). In large or multiple uterine fibroids, this treatment strategy is rarely used, especially in women who want to undergo uterine-preserving surgery (8). UAE can be a useful addition to surgery in women with massive fibroids (9). There are no studies on the use of preoperative UAE in combination with laparoscopic myoma enucleation in uterus myomatosus. The aim of the study was therefore to investigate the influence of the use of UAE before surgical laparoscopic myomectomy on the diagnosis of the uterus myomatosus and whether this combination therapy can meet the patient's desire for a uterine-preserving myomectomy.

## Materials and methods

### Data collection

The study is a retrospective, non-randomized, comparable clinical monocentric study with two comparison groups. A total of 202 patients were included. Of these, 101 patients who underwent surgery between February 1st, 2014 and March 31st, 2019 in the Clinic and Polyclinic for Gynecology and Reproductive Medicine at Jena University Hospital with the ICD-10 diagnosis of uterus myomatosus after preoperative uterine artery embolization (UAE) (first group). The comparison group also consists of 101 patients who underwent surgery between 2018 and 2020 in the Clinic and Polyclinic for Gynecology and Reproductive Medicine at Jena University Hospital with the ICD-10 diagnosis of uterus myomatosus without UAE (second group/control group). Local Ethics Committee approval is available (Reg. No.: 2021-2096 dates).

### Data management

All patient-related data was recorded in an anonymous form using assigned patient identification numbers. The study data was protected from unauthorized access and only employees of the study were allowed to access it.

### Statistical evaluation

The data were recorded in an Excel spreadsheet (Excel Version 2020. Microsoft, Redmod, USA). Then transferred to the statistical evaluation software SPSS (IBM SPSS Statistics Version 26, Chicago, Illinois, USA). Test-independent samples were used and *p*-values < 0.05 were considered statistically significant. The Welch *t*-test was used.

### Study hypotheses

The hypotheses of this study are: In the case of operative laparoscopic myomectomy in the uterus myomatosus, PUAE can shorten the duration of the operation, the hospital stay and the transfusion of blood supplies, increase the postoperative outcome and the overall satisfaction of the treated patients.

### Inclusion and exclusion criteria

#### Inclusion criteria

Age between 21 and 50, diagnosis of uterus myomatosus confirmed clinically, sonographically and with MRI. Symptomatic (bleeding disorders, pain symptoms) and asymptomatic fibroids in sterile patients for whom laparoscopic myomectomy has been indicated. As well as non-pedunculated submucosal, intramural, transmural, subserosal fibroids.

#### Exclusion criteria

Age below 20 and above 50 years, coagulation disorders, coagulopathies and vascular diseases, psychiatric illness that make study participation and follow-up questionable. Taking blood thinners, suspected sarcoma or malignancy, pedunculated subserous fibroids, pedunculated submucosal fibroids, intraligamentous fibroids. Localization outside the uterus, current pregnancy, myoma larger than 15 cm, infections in the urogenital tract, contrast medium allergy, hyperthyroidism.

All patients in the PUAE group received the following measures in addition to the normal course of inpatient admission: a preoperative 3 Tesla MRI of the pelvis in the radiology department of the University Hospital Jena (MAGNETOM Sola device from Siemens Healthineers Germany, Erlangen, Germany 2018), followed by an epidural anesthesia by the anesthetist, followed by embolization using Gelaspon under X-ray fluoroscopy with contrast medium 4 to 6 h before the elective laparoscopic fibroid enucleation by interventional radiologists from the Institute for Diagnostic and Interventional Radiology (IDIR).

In addition to epidemiological data, the following parameters were collected: symptoms (bleeding disorder, pain symptoms, feeling of pressure, sterility), gravida status, para status, menarche (year), BMI, secondary diagnoses, cycle length (days), duration of bleeding (days), bleeding intensity, pill intake, Previous therapy attempts, previous operations, size of the fibroids per cm (or the largest fibroid) measured by ultrasound and MRI, preoperative hemoglobin value (mmol/l), number of fibroids removed, location of the fibroids (submucosal, intramural, transmural, subserous, intraligamentous), operation date, operation duration (in minutes), postoperative hemoglobin level (mmol/l), intraoperative complications, postoperative hemoglobin level (mmol/l), postoperative complications, postoperative length of stay (days).

### Surgical therapy

The surgical therapy was carried out by qualified specialists in gynecology and obstetrics, certified by specialist societies and who have many years of experience in laparoscopy. The operative technique and the operative instruments were identical in both comparison groups. The patients were clinically examined by a specialist in a special consultation for fibroids and the indication for surgery was indicated by a specialist.

### Expiry of the UAE

The indication for preoperative myoma embolization took place in an interdisciplinary manner in cooperation with radiology and the clinic and polyclinic for gynecology and reproductive medicine in the myoma center of the University Hospital Jena. An inserted intrauterine device (IUD) had to be removed prior to UAE. On the day of admission, blood was taken, a peripheral intravenous indwelling cannula was installed, a urinary catheter was inserted and the peridural catheter was explained. The day after admission, the UAE was performed with PDK in the radiology angiography department. Under local anesthesia, the interventional radiologist places a five French catheter (Roberts uterine curve) in the right femoral artery, probing the left internal iliac artery and then the uterine artery using a microcatheter directed cranially. Advance the catheter into the artery supplying the uterus under constant X-ray control. Performing a selective angiography to illuminate the anatomy using an iodinated contrast medium. Then “free-flow” flushing (embolization) using 10–15 ml of Gelaspon by Bausch and Lomb, cut into 1 mm pieces, and dissolved in isotonic saline solution 0.9% Bc and contrast medium until hemostasis of the blood supply in the ascending responsible (ascending part of the uterus) segment of the left uterine artery. The catheter is then removed from the inguinal artery and a pressure bandage is applied to the patient, which remains in place for up to 6 h after the intervention. The patient underwent laparoscopic myomectomy 4 to 6 h later.

## Results

A total of 89 patients were included in the first group and 77 patients in the second group according to the exclusion and inclusion criteria listed in the Material and methods section. In the first group, the following intra- and postoperative complications were noted: a single intraoperative bowel injury and a single laparotomy. Here, an intraoperative conversion from a laparoscopic myomectomy to a laparotomy in suspected malignancy had to be made. This patient had to be excluded from the study. Thus, the complication rate in our study group was 1.12 % (1 of 89 patients) and corresponds to current statistical information on intraoperative complication rates (Danilyants 2020). In the second group, the following intra- and postoperative complications were noted. UAE-related complications (direct and indirect) were found in three patients. With the formation of a spur aneurysm of the femoral artery, one patient developed a post-puncture syndrome because of the epidural anesthesia and one patient suffered postoperative deep vein thrombosis. There were also non-UAE-related complications in the form of general surgical complications. A postoperative ileus due to an a.e. intraoperatively caused bowel injury. Thus, the overall surgical complication rate was 0.77 % (1/77 patients), as in the first group. The UAE-related complication rate was 3/77 patients, i.e., 2.31 %. No patient in either group underwent a hysterectomy intraoperatively or during the hospital stay.

### Analysis of the epidemiological/preoperative data

The mean age of the treated patients from the first group was 38.98 ± 6.045 years (mean ± standard deviation) and in the second group was 40.56 years ± SD of 6.269. No significant difference was found here.

The mean age at menarche in the first group was 12.82 ± SD of 1.395 years and in the second group 12.85 ± SD of 1.306 years. No significant difference was found here.

The mean number of fibroids in the first group was 2.33 ± SD of 2.194 fibroids and in the second group 2.70 ± SD of 2.189 fibroids. No significant difference was found here.

The average myoma size of the largest myoma to be measured preoperatively in the treated patients from the first group was 6.68 ± SD of 2.239 cm and in the second group 6.69 ± SD of 2.499 cm. No significant difference was found here. No significant differences were found in the distribution of the clinical symptoms in the two groups (lower abdominal pain, hypermenorrhea, anemia, feeling of pressure, height progression and desire to have children).

#### Pelvic pain

30% in the first group reported this symptom (27 out of 89 patients) and in the second group 40% (31 out of 77 patients). There were no significant differences at *p* = 0.181.

#### Hypermenorrhea

49% of the patients in the first group (44 out of 89 patients) and in the second group 64.9% (50 out of 77 patients) suffered from this symptom. There were no significant differences at *p* = 0.045.

#### Feeling of pressure

44.9% of the patients in the first group (40 out of 89 patients) and 36.3% of the patients in the second group (28 out of 77 patients) reported this symptom. There were no significant differences at *p* = 0.262.

#### Size progression of the fibroids

19.1% of the patients in the first group (17 of 89 patients) and 11.6% in the second group 11.6% (9 of 77 patients) reported a size progression. There were no significant differences at *p* = 0.190.

### Presence of preoperative anemia

7.8% of the patients in the first group (7 of 89 patients) and 12.9% in the second group (10 of 77 patients) had laboratory tests for anemia of grade 1 or higher. There were no significant differences at *p* = 0.278.

The mean BMI of the first group was 25.00 ± SD 4.858. The mean BMI of the second group was 24.50 ± SD 4.580. There were no significant differences.

In the first group, the intramural or transmural location of the fibroid was found in 57 of 89 patients, and in the second group, this location was found in 67 of 77 patients. There was no statistical significance at *p* = 0.251.

### Outcome analyses

The average operating time in minutes for the first group was 167.11 min. The mean operating time in minutes for the second group was 141.23 min. Thus, the operation lasted on average 25.88 min longer in patients who did not receive UAE prior to fibroid enucleation than in patients who previously received UAE. This results in a statistical significance of *p* = 0.0478 for the performance of the UAE before myomectomy in relation to the total duration of the surgery.

The average postoperative Hb value in the first group was 6.1 mmol/l. The average postoperative Hb value in the second group was 7.8 mmol/l. Thus, the mean postoperative Hb value of the first groups without a previous UAE intervention was 1.7 Hb points lower than in the patients who received a previous UAE intervention. A statistical significance with *p* = 0.0470 is shown in relation to the postoperative Hb value in favor of the second group, which received a UAE intervention.

A significant difference with *p* = 0.012 was found for the mean length of stay. On average, the length of stay was 0.5 days longer for patients in the first group compared to patients in the second group. The average length of stay of the treated patients in the first group was 5.21 ± SD of 1.458 days and in the second group 5.7 ± SD of 0.988 days.

To deepen the analysis, the patients were subgrouped within both comparison groups.

### Fibroid size over 6 cm

On average, 50.57% of patients (45 out of 89) in the first group had a fibroid size > 6 cm, and in the subgroup of the second group, 52.95% of patients (40 out of 77) had a fibroid size > 6 cm.

### Surgery time

The average operating time of the patients in the first group without UAE was 189.99 min. The mean operating time of the patients in the second group was 151.97 min. All patients had a fibroid ≥6 cm. On average, the operating time for the patients in the first group was 38.02 min longer than for the patients in the second group who had previously received a UAE and had a fibroid larger than or equal to 6 cm. A statistical significance is shown in favor of the second group with *p* = 0.0482. Thus, the preceding UAE prior to fibroid enucleation shows a direct benefit in terms of surgical time. A preceding UAE significantly reduces the operating time in the presence of large fibroids.

### Blood-loss

The average blood loss of the patients in the first group, who had no UAE and a fibroid larger than or equal to 6 cm, was 3.21 mmol/l. The average blood loss of the patients in the second group with UAE and a fibroid ≥6 cm was 0.02 mmol/l. Thus, a patient who did not receive UAE before fibroid nucleation and has a fibroid at least 6 cm in size has a 3.18 mmol/l higher Hb loss than a patient in the second group. This results in a statistical significance of *p* = 0.0471 in favor of the patients who received a UAE before fibroid nucleation and have a fibroid at least 6 cm in size.

## Discussion

When selecting the patients based on the exclusion and inclusion criteria, a total of 89 patients in the first group and 77 patients in the second group were included. No technical failure of the embolism was noted. No patient in either group underwent a hysterectomy. The following complications were observed in the patients of the first group. One patient in this group required a blood transfusion. The following intraoperative and postoperative complications were observed in the patients in the second group. In addition, UAE-related complications (direct and indirect) were found in this group: aneurysm spurium, post-puncture syndrome because of (PDA) epidural anesthesia. Deep vein thrombosis. General surgical complications of a postoperative thin closure due to an intraoperative bowel injury. In both groups, the surgical techniques and surgical instruments used were identical. In our study, complications in the overall context occur more frequently in UAE patients. The embolization-related complications described above as well as the general surgical complications. However, the inpatient stay of the UAE group was not longer than that of the non-UAE group. On the contrary, the UAE group showed a significantly shorter hospital stay of 0.5 days. So, it can be assumed that the complications that occurred in connection with UAE are to be rated as moderate and have no influence on the inpatient course. There were no significant differences between the two groups in terms of preoperative hemoglobin level and the number and weight of removed fibroids. In the study group, there was an average hemoglobin decrease of 1.4 g/dl postoperatively compared with 1.7 g/dl in the control group (*p* = 0.055). Blood transfusions were not performed in any case. The average duration of the operation was 136 and 120 min in the control group. Pathological changes in the pulsatility and resistance indices and complications that could clearly be attributed to the study intervention did not occur. There was a correlation between the weight and number of removed fibroids and the intraoperative blood loss. No significant difference in the hemoglobin drop, the intraoperative blood loss and the transfusion rate could be detected. This study was necessary to evaluate whether a significant difference in blood loss could be achieved in the comparison groups with uterus myomatosus (10). Like our study, a retrospective American study by Goldman et al. (11) also concluded that a UAE immediately before a laparoscopic fibroid eruption facilitates the surgical intervention, especially in the case of larger fibroids or large uteri. However, this study did not show a significant reduction in the duration of the procedure and could not minimize the intraoperative blood loss. The study evaluated data from 26 laparoscopic fibroid enucleations performed by the same surgeon between 2004 and 2010. Twelve patients were treated by UAE followed by laparoscopic myomectomy. The 14 patients in the control group were examined for age, calendar year, surgeon and number of fibroids removed. Surgical outcomes included preoperative clinical measurable uterine size, operative time, operative blood loss, and postoperative fibroid specimen weight. The data were analyzed using a 2-tailed *t-*test. The fourteen control patients underwent laparoscopic myomectomy alone. The UAE group had a larger mean preoperative clinical uterine size and a larger mean fibroid specimen weight measured postoperatively (595.3 vs. 153.6 g, *P* < 0.05) (11). In a study by David et al. (12), the use of UAE was presented in a very small number of cases. A total of three patients with a diagnosis of fibroids and a very large uterus weighing at least 1,100 g were included. The patient collective benefited from the combination therapy. A shorter inpatient stay as well as a shorter operation time and a significant reduction in intraoperative blood loss compared to the single implementation of conventional fibroid enucleation could be demonstrated (12). In 2018, Schnapauff et al. (13) the results of David et al. (12) with a larger patient collective of 21 patients. Patients who wish to preserve the uterus and suffer from large fibroids can benefit from this combination therapy c. The fibroid size of the patients in the study by Schnapauff et al. (13) was on average 12.7 cm. In one of the 21 patients, the administration of blood supplies was necessary. Another patient received a complete removal of the uterus (hysterectomy) intraoperatively despite the UAE. Eleven of the 21 patients could be examined postoperatively. Ten patients reported an improvement in their symptoms. Nine of the eleven patients would choose the treatment method again (13). Both studies included patients with a high uterine weight and/or large fibroids. The clinical picture of the uterus myomatosus was not included in either study. Current scientific findings on the use of combination therapy for UAE with conventional myomectomy are pending. The aim of this work was therefore to close this knowledge gap and to investigate the use of the combination therapy of UAE and conventional myomectomy in the ICD diagnosis of uterus myomatosus using the study designs described in detail in the Material and Methods chapter. The studies by David et al. (12) and Schnappauf et al. (30) served as the basis for this study. In contrast to these two studies mentioned, a higher number of patients were included in our study and compared with a comparison group with the same ICD-10 diagnosis using various parameters. In particular, the questions of intraoperative blood loss, operating times, length of stay in hospital and the postoperative patient outcome were examined. The working hypotheses of our study were detailed in the introductory chapter. In principle, we hypothesize that women who wish to have a uterus-preserving fibroid enucleation with the clinical picture of uterus myomatosus benefit from a combination therapy of UAE and conventional fibroid nucleation in that the intraoperative blood loss is less, the operation time is shorter, and the length of hospital stay is shorter compared to patients who receive a uterus-sparing fibroid enucleation without prior UAE.

To establish a new procedure in everyday clinical practice, various requirements must be met: the effectiveness and safety of the new method compared to previous methods, the additional costs and time required for the new method, as well as the availability of the experience of the hospital staff and the Quality of interdisciplinary collaboration between interventional radiologists and gynecologists (14–18). UAE requires several additional medical procedures, such as pelvic MRI, placement of epidurals, and performance of angiography, that are not performed with conventional laparoscopic fibroid enucleation (19). The experience of the radiologists and the availability of this method and the necessary equipment cannot be guaranteed in every hospital (20–23). The interdisciplinary myoma treatment in this way is demanding but can offer the option of having a positive influence on the surgical results if the indication is precise. Previous study results indicate that the combination therapy of UAE and myomectomy can be carried out easily in everyday clinical practice if the human and technical capacities are already available for this (24–28). In addition, the combination therapy of UAE and laparoscopic myomectomy represents a modern gynecological treatment method, which can demonstrably increase patient satisfaction and the postoperative outcome (29). In addition, this combined therapy method can in principle expand the spectrum of minimally invasive therapy in surgical gynecology for (very) large myomas, a high number of myomas and the clinical picture of the uterus myomatosus. The current trend toward minimally invasive and gentle surgical therapy methods, which are increasingly preferred by patients, can be strengthened with this combination therapy (30).

Other factors have expanded the established surgical method for removing fibroids in recent years. Such as the intraoperative use of tourniquet placing or administering tranexamic acid before surgery (14–16). What all methods have in common is the goal of reducing intraoperative blood loss during fibroid removal (17).

In addition to this fundamental trend development, with the combination therapy, the patient's desire for a uterus-preserving procedure in relation to fibroid therapy, even in the presence of large fibroids and/or many fibroids and the clinical picture of the uterus myomatosus can be promised and the statement by Scholz et al. (1) that the only therapy for uterine myomatosus is hysterectomy must be critically questioned.

## Conclusion

In summary, the study showed that the combination therapy of UAE with subsequent minimally invasive myoma nucleation can be a therapeutic option for the treatment of large myoma or the condition of uterus myomatosus, especially in patients who wish to have myoma removed after completion of the child wish due to possible complications in the medium and long term. By reducing intraoperative blood loss and shortening the surgery time, the UAE can improve the operating conditions and thus optimize the reconstructive capacity of the uterus from the surgeon's point of view. To date, very few studies have been established with many enrolled patients and a large number and size of fibroids. This study provides first findings in the field of minimally invasive myoma therapy in the treatment of large fibroids as well as in the clinical picture of the uterus myomatus in organ preservation procedures. It must be clearly mentioned that enormous costs and structural prerequisites must be met and that there are already optimized and common surgical treatment standards.

## Data availability statement

The original contributions presented in the study are included in the article/supplementary material, further inquiries can be directed to the corresponding author.

## Ethics statement

The studies involving human participants were reviewed and approved by University Jena. Written informed consent to participate in this study was provided by the participants' legal guardian/next of kin.

## Author contributions

SH conceived and planned the experiments and performed the analysis. IG and ES provided critical feedback and helped to shape the research, analysis, and manuscript. All authors contributed to the article and approved the submitted version.
